# Medical tourism's impacts on health worker migration in the Caribbean: five examples and their implications for global justice

**DOI:** 10.3402/gha.v8.27348

**Published:** 2015-04-10

**Authors:** Jeremy Snyder, Valorie A. Crooks, Rory Johnston, Krystyna Adams, Rebecca Whitmore

**Affiliations:** 1 Faculty of Health Sciences, Simon Fraser University, Burnaby, BC, Canada; 2 Department of Geography, Simon Fraser University, Burnaby, BC, Canada

**Keywords:** medical tourism, health human resources, global justice

## Abstract

Medical tourism is a practice where individuals cross international borders in order to access medical care. This practice can impact the global distribution of health workers by potentially reducing the emigration of health workers from destination countries for medical tourists and affecting the internal distribution of these workers. Little has been said, however, about the impacts of medical tourism on the immigration of health workers to medical tourism destinations. We discuss five patterns of medical tourism-driven health worker migration to medical tourism destinations: 1) long-term international migration; 2) long-term diasporic migration; 3) long-term migration and ‘black sheep’; 4) short-term migration via time share; and 5) short-term migration via patient-provider dyad. These patterns of health worker migration have repercussions for global justice that include potential negative impacts on the following: 1) health worker training; 2) health worker distributions; 3) local provision of care; and 4) local economies. In order to address these potential negative impacts, policy makers in destination countries should work to ensure that changes in health worker training and licensure aimed at promoting the medical tourism sector are also supportive of the health needs of the domestic population. Policy makers in both source and destination countries should be aware of the effects of medical tourism on health worker flows both into and out of medical tourism destinations and work to ensure that the potential harms of these worker flows to both groups are mitigated.

Medical tourism is a practice where patients travel abroad with the intention of accessing medical care, usually paid for by the medical tourist. Thus, it differs from cases of tourists seeking unplanned, emergency care while abroad and from cross-border care arrangements where insurers or public health systems reimburse their members for care received abroad. Although medical tourism is not a new practice, it has received increased public attention in recent years due to an expanding trend of residents of high income countries seeking care in low and middle income countries (1, 2). Although reliable numbers of the flows of medical tourists are not available, development of medical facilities serving medical tourists has expanded in recent years in well-established medical tourism destinations such as India, Thailand, and South Korea. Regional competition to attract medical tourists is also increasing, with medical facilities serving medical tourists opening or planned in the Middle East, the Caribbean, and Central America.

Medical tourism development is being sought primarily for the increased economic diversity and growth promised by this industry for destination countries (1, 3). It is also thought that medical tourism can stem the international out-migration of domestic health workers by providing them with relatively well-paying jobs and improved working conditions in the private health sector (4, 5). The retention of health workers could benefit other countries as well if increased local retention of workers reduces the need to seek health workers from other countries with health worker deficits (6).

For those health workers who have already emigrated in order to seek better pay and working conditions abroad, medical tourism is also seen as having the potential to lure these workers back to their home countries. In Malaysia, for example, the government has specifically targeted its nationals working abroad for employment in the domestic medical tourism sector (7). The return of these workers may increase the quality of care in the domestic health system, especially if workers returning home after training abroad introduce new skills and medical expertise into the domestic system (6, 8).

However, this optimism around the positive impact of medical tourism on health human resources is matched by concern that medical tourism creates pressures on public health systems in destination countries by encouraging health workers to opt for employment in the private rather than public sector. These effects have been documented in established medical tourism destinations including Thailand (4, 9, 10), Malaysia (11, 12), and India (13, 14). In Thailand, a requirement that medical practitioners speak Thai has resulted in heavy recruitment of Thai nationals from the public sector to fill domestic medical tourism employment (7). Movement by Indian health workers from the public sector to the private, medical tourism sector has been linked to concerns about patients in the public sector relying on less qualified health workers as a result of this movement (13). Moreover, medical tourism in India is indirectly subsidized by public resources used in training health workers who then go on to work in private medical facilities serving medical tourists, generating little to no direct benefit for the domestic population of public health system users (15). Existing shortages among specialists in the Indian public sector could be exacerbated because these workers will be in high demand in the medical tourism sector (16).

If, as might be expected, the movement of health workers to private health systems worsens working conditions for health workers in public health systems (i.e. by increasing the patient-to-worker ratio), then a vicious cycle may emerge in which deteriorating working conditions encourage increased health worker movements (17). Furthermore, when middle-income countries face health worker shortages as a result of medical tourism, they often turn to neighboring countries for health worker recruitment (18). Patterns of internal migration driven by increased medical tourism may also include rural to urban migration, which is particularly troubling because it shifts health workers away from often already underserved areas and further undermines health equity *within* countries (19). The impacts of medical tourism's redistribution of health workers may be felt in the training priorities of these countries as well. For example, both government and student training priorities might shift to the needs of the more lucrative private medical tourism market, leading to fewer health workers being trained according to the needs of the domestic population and public system (6). Research on the health equity impacts of medical tourism is still in its early stages, and much remains unknown about the impacts of this industry on health human resources in destination countries (3).

A second, less discussed impact of medical tourism on health worker migration is to encourage the migration of non-citizen health workers to medical facilities serving medical tourists abroad. Medical tourism-driven health worker migration is necessary in countries with small populations or limited health worker pools that cannot support the number of health workers and range of specialists needed to staff a large medical tourism facility. Several Caribbean nations fit this description and have been seeking to establish new medical tourism facilities that would explicitly rely on migrant workers to some degree to meet the staffing needs of these facilities. These countries have been actively competing with one another for foreign investment in the medical tourism sector, giving investors considerable leverage in shaping the terms of sector development. Prior to finalization of a new medical tourism development in Grand Cayman, for example, the local government made numerous legislative changes aimed at encouraging this investment, including the relaxation of licensure requirements for health workers, despite strong opposition from local health workers (20). This dimension of the interrelationship between medical tourism, health worker migration, and trade in health services has been little discussed in the academic literature on these topics. Because this type of health worker migration has a range of potential negative impacts on health worker training, health worker distribution, health system users in the source and destination countries for these migrants, and ultimately health equity, there is great need to better understand this phenomenon.

We have observed multiple distinct patterns of medical tourism-driven health worker migration to medical tourism destinations during our extensive fieldwork in the Caribbean. This fieldwork includes informal discussions and formal interviews with over 200 medical tourism stakeholders across Belize, the Bahamas (21), Barbados (6, 22–25), the Cayman Islands (26), Jamaica (26), St. Lucia (27, 28), and Trinidad and Tobago (29) since 2011, as well as extensive tours of formal and informal health-care facilities in these countries and visits to the sites of intended medical tourism clinics. We do not provide details on the methods and purposes of these various studies in this paper. Instead, we offer full references to articles that document our fieldwork, data collection, and site visits. In this paper we draw across observations made in our fieldwork, issues raised by policymakers and health workers we have met in these countries, and our continued following of local news and policy reports about the development of the medical tourism sector in the Caribbean to present a conceptual framework of health worker movements. We provide real-world examples of each of these movements in order to present ‘case studies’ of each movement that have been brought to our attention throughout the course of our research involvement in the Caribbean. The cases we discuss here are meant to be illustrative of particular movements in order to develop the framework of the five migratory patterns presented herein; and although we present an exhaustive listing of the patterns we have learned of there are indeed many other cases of each that are not shared here for the sake of brevity.

In the remainder of this paper, we discuss five distinct patterns of medical tourism-driven health worker migration experienced in the Caribbean region that we have gleaned from our fieldwork by drawing on specific case studies to provide ‘real-world’ examples of these mobilities. We also draw out the implications of this migration for global justice and potential policy responses. Although the impact of medical tourism on health worker migration has been discussed elsewhere (30, 31), this paper is the first to identify and discuss specific patterns of health worker migration, informed by direct observation and first-hand insights, to an *emerging* regional medical tourism export market.

## Five patterns of medical tourism-driven health worker migration

In this section we discuss five patterns of medical tourism-driven health worker migration: 1) long-term international migration; 2) long-term diasporic migration; 3) long-term migration and ‘black sheep’; 4) short-term migration via time share; and 5) short-term migration via patient-provider dyad. To identify these patterns we undertook a three-step process: 1) we independently summarized patterns that had been brought to our attention throughout the course of our fieldwork and in our ongoing media and policy reviews; 2) we met face-to-face to share what we had independently identified, reaching consensus on five distinct patterns; and 3) we came to agreement as to the cases that best serve to illustrate each pattern based on our experience in the Caribbean region, focusing on ones that had media reporting or other documentation that could be drawn upon to support their characterization herein.

### Pattern 1: Long-term international migration

The first approach to addressing the staffing problem of large medical tourism facilities in destinations unable to meet their needs is to allow the long-term migration of health workers to fill these positions. This is the method by which the Health City Cayman Islands development is addressing the considerable staffing demands of its new facility. The first phase of this development opened in February 2014. It includes 140 beds focusing on cardiovascular disease and orthopedic surgery (32). Additional phases of this project are planned and will include a 2,000-bed multi-specialty hospital and a medical school that will train new health workers from both the local population and abroad (32, 33). The Health City project is targeting a diverse range of customers but because of its large size cannot rely solely on the domestic population as a customer base. Rather, it will be looking to both regional and North American markets for customers as well (33, 34).

The facility plans to attract patients by competing on cost with procedures offered in its customers’ home countries (32, 33). This lower procedure cost is made possible by the relatively lower labor costs of the health workers staffing the facility, low medical malpractice liability, and a pioneering approach to surgery developed by Health City's Indian founder, Devi Shetty. Shetty's Narayana Hrudayalaya chain of hospitals in India function as an ‘assembly line’, completing a large volume of surgeries with surgeons and other health workers focusing on a discrete task repeated on each patient (32). In order to transfer this care model to the Cayman Islands, Shetty is importing 70 Indian doctors, technicians, and nurses for the Health City development (33). Presumably these numbers will increase if and when the later and much larger stages of the hospital are completed. These health workers are complemented by the local population, who have been recruited to provide administrative and support staff (33). Shetty has stated that the Health City development will staff 25% of its overall workforce with Caymanian citizens over the long term (32).

### Pattern 2: Long-term diasporic migration

Medical tourism is promoted as potentially encouraging the return of domestic health workers who have migrated abroad to practice. For some medical tourism facilities, drawing on the diaspora of migrants from the destination country is the facility's explicit business model. This is the case with American Global MD, a network of US-trained doctors that plans to break ground on a US$170 million dollar medical tourism facility in Jamaica in 2015 (35). American Global MD has marketed itself as having members with a connection to Jamaica, described as a ‘consortium of American-trained physicians and investors, all of whom have Jamaican links as a result of having previously studied or practiced in the country’ (35, 36). The numbers of US-trained physicians likely to take part in this project have not been given. Some of these workers will staff the planned hospital, but it will also rely on local doctors, nurses, and technicians. The first phase of the proposed 50–75-bed hospital will focus on cosmetic, bariatric, and dental services. In future years, a 200-bed multi-specialty hospital is planned. As with Health City Cayman Islands, the American Global development will target domestic, Caribbean, and North American markets on the basis of cost with domestic facilities in these markets, especially in the United States (35).

### Pattern 3: Long-term migration and black sheep

Investors seeking to develop medical tourism facilities are often able to shop or lobby for regulatory environments that are the most accommodating to their plans and needs. In the Caribbean and elsewhere, this accommodation has allowed developers and individual health workers to operate facilities that would not be permitted in jurisdictions with more stringent regulations. Because some countries are extremely eager to participate in the perceived medical tourism ‘gold rush’ now taking place, they have been willing to relax regulatory protections to attract these medical tourism black sheep to their countries. Many examples of these black sheep exist, including Peter Nygard, a 70-year-old Finnish-Canadian fashion designer and stem cell enthusiast. Nygard, a resident of the Bahamas, is actively promoting plans to invest in a new stem cell clinic in the Bahamas staffed by foreign health workers that he will recruit to this project (37). Toward this end, he has proposed new legislation there to allow experimental stem cell treatments in humans (38). His involvement in legislative changes in the Bahamas has been controversial, with the Prime Minister of the Bahamas denying Nygard's involvement amid accusations of undue influence by Nygard (39). Other forms of black sheep medical tourism exist as well, including the migration of individual health workers who are fleeing trouble at home. For example, a US-trained cardiac surgeon who migrated to Barbados to establish a cardiology clinic targeting international patients there has been mired in controversy around his local licensing following the revelation that his US medical license was revoked due to a sexual assault conviction (40).

### Pattern 4: Short-term migration via time share

As opposed to the long-term health worker staffing patterns discussed in the previous examples, some medical tourism sites in the Caribbean have opted for solutions that rely on short-term movements by health workers. One such migratory pattern entails health workers practicing abroad on a ‘time share’ model where they purchase the right to be the only individual offering a service for patients from a specific geographic region (typically where their main practice is based) and the right to practice for part of each year in an off-shore medical facility. The American World Clinics facility proposed for Barbados seeks to operate on this model (41). American World Clinics plans to open a 60-bed multi-specialty hospital on the site of a former private hospital (42). Physicians staffing the hospital will be drawn primarily from the United States, and American World Clinics has already hosted several events in Barbados for physicians interested in buying into the program. Nurses, technicians, and other workers will largely be staffed from the domestic population (43). This facility will target the local, regional, North American, and European markets and will compete on cost (43). American World Clinics sees the relative laxness of regulatory protections in Barbados (which they have in part negotiated for) as helping to reduce costs and also as allowing experimental procedures and research that would not be possible in the United States or European Union (44). The Barbados development has been delayed pending issues around financing and loan guarantees, and subsequently American World Clinics has expressed interest in pursuing development in the Bahamas, Grand Canary, Costa Rica, and the Dominican Republic (45).

### Pattern 5: Short-term migration via patient-provider dyad

Whereas the time-share model of medical tourism-driven health worker migration has health workers moving to another country to practice for part of each year (Pattern 4), a final pattern of migration consists of health workers traveling abroad with their patients for the duration of their treatment. This patient-provider dyad repeats, with health workers traveling with each new patient or group of patients to the same destination. An example of this pattern of migration is Canadian surgeon Jim MacKenzie, who accompanies patients to and from the Turks and Caicos where he performs orthopedic surgeries (46). These patients are motivated to travel abroad for surgery by wait times for their care in Canada and are able to access this care more quickly by opting for private international treatment. Canadian doctors cannot provide essential medical services in Canada outside of the public system, but MacKenzie and other Canadian doctors can provide their patients with pre- and post-operative care in Canada, paid for by the public system, while being compensated out-of-pocket for the surgery taking place in the Caribbean (46).

## Implications for global justice

These five patterns of medical tourism-driven health worker migration have important implications for global justice, understood as a concern with the distribution of goods essential to human life (such as health care) and the structure of the political systems that govern this distribution. Although theories of global justice vary, when a global institution such as medical tourism systematically disadvantages some and benefits others in terms of their access to health care, this pattern of distribution is a matter of concern to global justice (1).

A first implication for global justice focuses on the impacts of medical tourism on health worker training. The migration of health workers to the Caribbean is commonly viewed as creating new opportunities for training and skills exchange for local health workers. For example, the medical school planned as part of the Health City Cayman Islands project, if realized, could provide new training opportunities for Caymanians and, over the long-term, help the Health City development place more Caymanians in specialist positions (47). Jamaican health workers that trained in the United States are able to bring with them training and experiences that may not be available in Jamaica, potentially leading to new training opportunities and exposure to new medical techniques for local health workers. The presence of US-trained health workers in the American World Clinics facility carries similar potential for the local population, though such opportunities are not a stated goal of the project and may not be realized (43). Finally, the regular visits by the same team of doctors to the same facilities in the Turks and Caicos may expose local health workers to new training opportunities and techniques, though the temporary nature of these visits is likely to disrupt these opportunities (48).

Although there is some promise of exposure to new training opportunities for local health workers as a result of new medical tourism investment, these patterns of migration are also potentially disruptive to domestic training activities. This is particularly true of the short-term and less stable patterns of migration, where migrants are unlikely to be part of a sustained and centrally planned training effort. In the case of Barbados, relationships between visiting physicians and local staff are likely to be unstable, making it difficult to develop sustainable training opportunities for local health workers. Similarly, the temporary nature of the movement of international health workers in the Turks and Caicos model puts the value of training opportunities for destination countries in doubt.

The medical school planned as part of the Health City Cayman Islands project is more likely to provide organized and sustainable training opportunities for domestic health workers; however, this project raises the additional concern that even when training activities are sustainable they are likely to be driven by the needs of the private, medical tourism industry. That is, rather than developing new techniques for addressing the primary health needs of the domestic population, these training opportunities will serve to subvert local priorities, preparing domestic health workers for work in facilities that focus on tertiary care in a private setting. The long-term effects of this co-option of domestic training programs could be profound, shifting the training of generations of health workers away from the priorities of their compatriots, a concern that has been echoed in medical aid missions abroad (49). Health City Cayman Islands relies on a highly specialized and very narrow model of health-care provision, and if the planned medical school follows this model then graduates might have difficulty obtaining positions outside of the local medical tourism system. The assembly-line approach of care developed by Shetty may be appropriate for the vast population and needs in India, but it is less clear if this culture of care is appropriate for a small Caribbean island of under 60,000 people (50).

A second implication for global justice focuses on medical tourism's impact on health worker movements. The patterns of health worker migration in the above cases are promoted as reversing or reducing the out-migration of domestic health workers from medical tourism destination countries in the Caribbean. This potential is clearest in Jamaica, where the proposal to draw on expatriate health workers could create a stable, long-term workforce. Because these workers have ties to Jamaica, their integration into the local health system is likely to be more sustainable and they are more likely to integrate into local communities than the workers discussed in the other patterns. This migratory pattern has the advantage of addressing domestic health human resource needs with workers who are linked culturally with the home country to which they are migrating. As such, they are likely to be more attuned to the needs of the local population and able to fit within that country's culture of care. In the other cases, new, higher paying jobs linked to training opportunities in the private sector are thought to help reduce the pull of jobs in other countries, thus reducing the emigration of health workers from the Caribbean.

That said, these patterns of health worker migration also have the potential to enable and reinforce problematic ongoing patterns of migration, where nations losing health workers to international migration turn to other nations to recruit replacement workers. As the above patterns of migration demonstrate, a range of countries are potential sources of health workers for the medical tourism industry, including middle income countries like India and high income countries like Canada and the United States. If the losses of health workers in these countries are sufficient to create new health human resource needs, these countries may turn abroad to recruit new workers, reinforcing a cycle of recruitment and migration driven by the private health-care sector. Similarly, if Caribbean countries find their domestic workers drawn into the private, medical tourism sector, they too may turn to regional countries in order to address their health worker needs in the public sector. For example, Jamaica has sought to address its existing health worker needs by recruiting health workers from Nigeria, Ghana, and Guyana, which contributes to health worker shortages in those countries (51). If health worker shortages in the public sector are exacerbated by demand in the private, medical tourism sector in the future, recruitment from countries with health worker shortages will continue and potentially worsen existing global injustices.

A third implication for global justice focuses on medical tourism's impacts on local health care. Medical tourism is promoted as having the potential to expand the range and potentially the quality of medical services available to locals. For those able to afford access to the proposed American World Clinics facility, the range of services available in Barbados will increase greatly as a result of the opening of this facility. Similarly, the presence of a multi-specialty hospital in Cayman will allow the local population to access a wider variety of care and likely improved quality of care without having to go abroad themselves as medical tourists, as is often now the case (32). This expansion of services may include access to experimental procedures as well, as in the case of residents of the Bahamas.

Despite these potential benefits, it is not clear that the health workers being recruited as part of medical tourism initiatives will be well placed to meet the needs of the local population. One concern is that migrating health workers will not be familiar with local cultures of care, health systems, and population health needs. In Barbados, because the international physicians are not relocating to Barbados permanently, they are likely to be less familiar with the local culture and particularly the culture of care, potentially disrupting practices in the domestic health system (though disrupting problematically hierarchical or inefficient practices could be beneficial). Another concern is that the introduction of migrant workers serving the needs of international patients will crowd out the domestic population from local facilities. In the Turks and Caicos, the use of existing facilities by relatively wealthy foreigners carries the potential of crowding out local health workers from using these facilities and disadvantaging the local population. In Jamaica, recruitment of diaspora workers is taking place on the basis of their connections to Jamaica rather than their qualifications. Therefore, these health workers may not be the best qualified and may not be those that Jamaica would seek to recruit were it focused solely on the needs of the local health system. Although it is possible that the training these workers have received will be of higher quality than that available locally, it is not clear that this training will be locally appropriate, taking into account the health needs and priorities of the local population and the infrastructure in place to meet these needs. Most importantly, these workers are being recruited primarily to serve the needs of international patients rather than the domestic population. Although locals may access the planned facility by paying out of pocket to do so, the services being offered are not shaped by the needs of most Jamaicans but by the needs of international customers with the ability to pay privately for health services.

A final implication for global health is the impact of medical tourism on local economies. Medical tourism is promoted as benefitting local populations through economic growth that will benefit the entire populace. In many cases, these small island nations are highly dependent on the traditional tourism industry and so are highly vulnerable to changes in the economies of larger nations on which they depend for customers. By diversifying their export base, these countries may encourage economic growth and be less vulnerable to global economic downturns.

However, government support of these facilities creates new financial risks throughout the destination countries. Experimental medical procedures such as those in the Bahamas create particular dangers for the local population. As these developers and practitioners are fleeing tighter regulations and other restrictions at home, there is a danger that their new destination countries will be stuck with problematic procedures and personnel over the long term. These migrants can harm their patients and also cause reputational harm to their destination countries. This was the case in Barbados, where a stem cell clinic that injected patients with stem cells from aborted fetuses without their patients’ knowledge was the target of an expose by the BBC (52). In other countries, the resources needed to lure foreign investment in the medical tourism sector may not be recouped, creating costs for the domestic population. These costs include the following: financial outlays such as tax breaks, land leases, and trade missions; loss of human resource time spent promoting and planning for medical tourism development; and alterations to the local health system, such as regulatory changes to malpractice laws (53). Each of these changes can disadvantage the local population, losses that may not be made up for by a successful medical tourism sector.

In many respects, health worker migration and medical tourism are two sides of the same coin, entailing the international movement of health workers on one side and the international movement of patients on the other. The patterns of health worker migration described in this paper show that medical tourism serves to drive this migration and to promote fluidity of the global health worker supply, enabling workers to have access to the most lucrative and personally rewarding workplaces. Although this phenomenon creates new opportunities for these workers, it does so in ways that can be problematic for health equity and global justice. This fluidity has the effect of undercutting domestic control over the training and distribution of these workers and promotes the redistribution of workers from the public to the private sector. By lowering barriers to the movement of patients and health workers, medical tourism privileges those able to take advantage of new opportunities in the international trade of health services, disadvantaging those unable to access these services and left behind in their domestic health systems.

## Policy responses

In light of the potential negative effects of medical tourism-driven health worker migration, several policy responses are available to different stakeholder groups in the destination countries for these medical tourism facilities. As was observed in the above examples of five unique migration patterns, medical tourism developers typically seek concessions from potential destination countries that include liberalizing licensure requirements in order to accommodate the international health workers that they wish to use to staff their facilities. These concessions subvert the role of licensure, directing it toward the facilitation of medical tourism developments that serve relatively wealthy foreigners and locals rather than protecting the health and safety of the majority of the local population. Existing licensure requirements may benefit from modification, of course. Health worker licensure can be used for protectionist ends, shielding local practitioners from competition and leading to the problem of ‘brain waste’, where well-qualified health workers with foreign credentials are denied an opportunity to practice in the jurisdictions to which they have migrated (54, 55). That said, local health professional groups have a role in using their influence around licensure requirements to protect the interests of those accessing the local health system and to resist changes to licensure that will weaken patient protections in the interest of outside groups.

In order to resist co-option of domestic training opportunities for the purposes of the medical tourism sector, local health worker administrators should use their role in overseeing training and accreditation to push for new opportunities that are locally relevant and sustainable. This can be achieved by monitoring degree and licensure requirements for local graduates of training programs and engaging actively with the directors of medical facilities serving medical tourists to shape the opportunities being offered to local health workers.

A general concern with the entry of medical tourism developers into new markets is that legislative and policy changes made to support this industry will harm the domestic health system and its users. In some cases promotion of the medical tourism sector is presented as a trade-off, where concessions are needed in order to attract investment that will diversify the local tourism product and provide new employment opportunities for locals (20). In the Caribbean, solicitation of foreign investment into the medical tourism sector is typically led by Ministries of Tourism and public-private investment promotion bodies such as Invest Barbados and Trade and Investment Jamaica (6, 43, 53). These bodies are well intentioned, seeking economic development for their home countries, but they may not be familiar with the potential impacts of medical tourism development on their home health systems or be prepared to represent domestic health interests when negotiating with foreign investors. Therefore, local Ministries of Health must be integrated into medical tourism sector promotion and planning from the start in order to ensure representation of the interests of domestic health workers and health system users. In this way, long-term planning for domestic health sector needs can be part of the strategy for medical tourism sector development, making it more likely that the promised benefits of these developments to health worker training and distribution will materialize.

Finally, source countries for migrant medical tourism sector health workers have a role in responding to these patterns of migration as well. Although the numbers of health workers being lost in countries such as India, Canada, and the United States are thus far modest, there is a potential that medical tourism-driven health worker migration could create a long-term drain on health human resources in these and other countries. Policy responses for these countries are limited in that it is practically difficult and potentially ethically problematic to restrict the movement of health workers who wish to practice abroad (55, 56). At the very least, however, these countries should be aware of these growing patterns of movement and work to track the numbers of migrants and the impacts of these movements on domestic health systems. More concretely, these countries should explore widening the scope of the World Health Organization Global Code of Practice on the International Recruitment of Health Personnel to address these migratory patterns (57). Unless source countries are aware of the size and impacts of these patterns of migration, they will not be able to develop timely policy responses, including adjusting training programs and addressing domestic working conditions. Advance planning will make it more likely that these countries are able to respond to the loss of health human resources from within their own countries rather than having to rely on the damaging cycle of recruitment of health workers from other countries.

## Conclusions

Medical tourism is a global health services practice that involves the movement of patients across national borders in search of private health care abroad. Whereas the bulk of the medical tourism literature is focused on issues and concerns surrounding these patients, here we have shown that health workers too are moving temporarily and permanently as part of this international industry and thus also warrant consideration. We identified five unique patterns of health worker movements associated with the growing medical tourism sectors in the Caribbean: 1) long-term international migration; 2) long-term diasporic migration; 3) long-term migration of black sheep providers; 4) short-term international migration via a time share model; and 5) short-term migration of the patient-provider dyad. Although each pattern is distinct, they together demonstrate some potential harms to local health systems and health system users resulting from such movements, including generating limited gains to local patients and medical trainees. These harms hold implications for global justice and health equity more broadly because they may enhance health worker numbers in areas not relevant to local health system needs, as but one example.

As a way to counteract harms, we recommend that policy initiatives be undertaken so as to ensure that citizens, health workers, and health systems in medical tourism destination countries at the very least do not lose out as a result of local participation in this global practice. Herein we propose initiatives such as involving local health worker professional bodies in regulatory decision-making, ensuring new training opportunities for local health workers that draw on the expertise of migrant health workers practicing in medical facilities serving medical tourists, and ensuring that health-sector representatives are centrally involved in all policy and regulatory developments pertaining to medical tourism. Although our focus here has been on the Caribbean, drawing on specific examples from the Cayman Islands, Jamaica, the Bahamas, Barbados, and Turks and Caicos in particular, the patterns of movement we have identified are happening elsewhere. Thus, the policy responses suggested here, in fact, contribute to a global dialogue centered upon discerning the harms and benefits associated with this industry, so as to bring forth evidence that can inform a more equitable medical tourism practice (1, 3, 6, 8).
